# Editorial: Contemporary strategies: advancing healthcare for HIV, STIs, and beyond

**DOI:** 10.3389/frph.2023.1259732

**Published:** 2023-08-04

**Authors:** V. A. Edward, A. A. Chuturgoon, S. T. Lalla-Edward

**Affiliations:** ^1^ The Aurum Institute, Johannesburg, South Africa; ^2^ School of Health Sciences, College of Health Sciences, University of KwaZulu-Natal, Durban, South Africa; ^3^ Department of Environmental Health Sciences, Yale School of Public Health, New Haven, CT, United States; ^4^ Department of Medicine, Vanderbilt University, Nashville, TN, United States; ^5^ School of Laboratory Medicine and Medical Sciences, University of KwaZulu-Natal, Durban, South Africa; ^6^ Ezintsha, Faculty of Health Sciences, University of the Witwatersrand, Johannesburg, South Africa

**Keywords:** mHealth, machine learning, self-testing, community pharmacy, HIV risk, self-care, cardiovascular risk, community based primary health care

**Editorial on the Research Topic** Contemporary strategies: advancing healthcare for HIV, STIs, and beyond

A crucial component of universal health coverage, is the promotion of sexual and reproductive health. The various dimensions which contribute to this particular health right being actualised, include disease screening, disease prevention, health management, education, and counselling services (1). As a result, sexual and reproductive health interventions require integrated participation across several levels; from individual to policy levels (2, 3). This collection of articles facilitates reporting of some of the key issues pertaining to HIV, sexually transmitted infections, maternal health, and health systems, with a particular focus on emerging technologies and strategies to expedite the provision of appropriate health solutions.

Whether accessing HIV care and viral suppression can be achieved by offering financial incentives to patients with HIV is elucidated upon by Majam et al. Describing original pilot research that they conducted in Johannesburg, South Africa, the authors explain the extent to which participants used smartphone applications to share their HIV related results, and the usefulness of financial incentivisation as a mechanism for engagement in care (Majam et al.). A similar pilot study, this time exploring the sole use of mHealth in HIV intervention, tested how amenable participants were to responding to short message service reminders and interactive voice response system communication to report their HIV status after self-testing (Gaven et al.). Another strategy that is gaining momentum, is the use of differentiated service delivery to expand access to health services and required medication. The role of community pharmacists and community pharmacies as a potential platform for HIV products and services is considered in *The South African community pharmacy sector—an untapped reservoir for delivering HIV services* (Nyamuzihwa et al.). The authors describe the proliferation and distribution of community pharmacies, how the role of the community pharmacist can be expanded and why this presents a viable opportunity to contribute to HIV mitigation and attainment of UNAIDS targets.

The path of HIV self-testing from its conception over 25 years ago to its current iterations, is traced by Fischer et al. They highlight the salient steps and features in HIV self-testing and discuss the issues that arise from packaging digital interventions with HIV self-testing. Given the momentum that such interventions are gaining, data management is a growing concern, impeding data harmonization and scalability. The authors therefore raise the question of whether there is a need for regulatory approval or prequalification of digital interventions used with HIV self-tests (Fischer et al.). The role that technology can play in HIV screening and sexual health matters among men who have sex with men and gender diverse communities, is reported on in two other articles. Abraham et al. investigated the disposition of members of the aforementioned marginalised communities in Australia when presented with the option of accessing sexual health services on digital applications and web based channels (Abraham et al.), while Zhao et al. in their Chinese study probed the potential of establishing online HIV testing platforms on a dating site. They identified drivers and obstacles to app-based HIV testing services at various levels (Zhao et al.). Both these studies aimed at mitigating common barriers to HIV testing, like fear of stigmatisation and accessibility of services. A different technological advancement which enters the fray, is *The role of machine learning in HIV risk prediction* (Fieggen et al.). This perspective article clarifies what machine models are, and how they are developed and evaluated. The authors make a case for including machine learning as an HIV prevention tool of the future, and discuss the benefits of the predictive quality of machine learning in identifying at risk individuals, amongst its various other uses. Keeping with risk, Adedokun et al. recommend routine risk screening for cardiovascular diseases since their Nigerian study found that sub-clinical atherosclerosis was higher in HIV treatment experienced patients, and this was irrespective of the presence of traditional risk factors (Adedokun et al.).

The need to address social and systemic factors that impact sexual and reproductive health initiatives is examined in the remainder of the articles. The Maharaj opinion piece that adolescent pregnancy in sub-Saharan Africa is concerning, draws attention to the reasons for this as well as highlights some interventions which have been successful in curbing the adolescent pregnancy rate in other countries. The authors advocate for greater involvement from all sectors of society in minimising the adolescent birth rate (Maharaj). Another at risk sector is identified in *Enrollment and retention of female sex workers in HIV care and health facilities in Mabara city.* Readers are reminded that the sexual health needs of sex-workers often go unattended despite them being very much in need of such. In this original study, among various themes, the impact of healthcare workers’ attitudes and the quality of services at health facilities on female sex workers’ motivation to enter into HIV treatment is discussed (Arinaitwe et al.).

South Africa finds itself in the spotlight in another two articles, where attention is drawn to the country’s shortfalls but accompanied by suggestions for improving the health systems. Using South Africa as an example of low- and middle- income countries in general in their assessment, Ordonez et al. explain the link between colonialism and a sub-standard healthcare system. They illuminate the need for innovation and a pragmatic integrated approach to addressing the HIV healthcare services in neo-colonial South Africa (Ordonez et al.). While the final perspective article by Nyatela et al. also provides advice to various stakeholders, they focus on how the identified stakeholders can support patients as they engage in self-care. The authors reflect on the lessons learned from the COVID-19 pandemic, and use them as a foundation on which to improve HIV mitigation generally and especially as they pertain to patients with co-morbidities (Nyatela et al.).

This collection of original research, perspectives and opinion articles provides a holistic overview of the different approaches to research into HIV and sexual reproductive healthcare. What becomes clear is that the commitment to delivering effective healthcare remains, and that innovative approaches to the provision of such continue to evolve.
